# Investigating Fractal Analysis as a Diagnostic Tool That Probes the Connectivity of Hippocampal Neurons

**DOI:** 10.3389/fphys.2022.932598

**Published:** 2022-06-23

**Authors:** Conor Rowland, Bruce Harland, Julian H. Smith, Saba Moslehi, John Dalrymple-Alford, Richard P. Taylor

**Affiliations:** ^1^ Physics Department, University of Oregon, Eugene, OR, United States; ^2^ School of Pharmacy, University of Auckland, Auckland, New Zealand; ^3^ School of Psychology, Speech and Hearing, University of Canterbury, Christchurch, New Zealand; ^4^ New Zealand Brain Research Institute, Christchurch, New Zealand

**Keywords:** neurons, connectivity, fractal analysis, fractal dimension (D), modified Sholl analysis, neuromorphology, hippocampal CA1, anterior thalamic nuclei

## Abstract

Many of nature’s fractal objects benefit from the favorable functionality that results from their pattern repetition at multiple scales. Our recent research focused on the importance of fractal scaling in establishing connectivity between neurons. Fractal dimension *D*_*A*_ of the neuron arbors was shown to relate to the optimization of competing functional constraints—the ability of dendrites to connect to other neurons versus the costs associated with building the dendrites. Here, we consider whether pathological states of neurons might affect this fractal optimization and if changes in *D*_*A*_ might therefore be used as a diagnostic tool in parallel with traditional measures like Sholl analyses. We use confocal microscopy to obtain images of CA1 pyramidal neurons in the coronal plane of the dorsal rat hippocampus and construct 3-dimensional models of the dendritic arbors using Neurolucida software. We examine six rodent groups which vary in brain condition (whether they had lesions in the anterior thalamic nuclei, ATN) and experience (their housing environment and experience in a spatial task). Previously, we showed ATN lesions reduced spine density in hippocampal CA1 neurons, whereas enriched housing increased spine density in both ATN lesion and sham rats. Here, we investigate whether ATN lesions and experience also effect the complexity and connectivity of CA1 dendritic arbors. We show that sham rats exposed to enriched housing and spatial memory training exhibited higher complexity (as measured by *D*_*A*_) and connectivity compared to other groups. When we categorize the rodent groups into those with or without lesions, we find that both categories achieve an optimal balance of connectivity with respect to material cost. However, the *D*_*A*_ value used to achieve this optimization does not change between these two categories, suggesting any morphological differences induced by the lesions are too small to influence the optimization process. Accordingly, we highlight considerations associated with applying our technique to publicly accessible repositories of neuron images with a broader range of pathological conditions.

## 1 Introduction

Fractal objects are abundant in nature because they allow optimal patterns to be repeated over multiple scales (Bassingthwaighte et al., 1994; Iannaccone and Khokha, 1996; Frame and Urry, 2016). The dendritic arbors of neurons are fractal tree-like structures that contribute to complex neural circuits within the brain. These arbors are highly dynamic structures that influence how the neuron receives and processes synaptic inputs. Brain disorders are associated with alterations in neuronal morphology suggesting the importance of dendrite integrity, including microstructural relationships, on the overall functioning of neuronal networks (Kulkarni and Firestein, 2012; Herzog et al., 2020).

A number of simple metrics have been used to quantify dendritic arbor morphology such as total length of dendrites and total number of branch points (Uylings and van Pelt, 2002). For example, dopamine denervation in a Parkinson’s disease mouse model resulted in reduced dendritic branch length and number of branch points in D1 and D2 medium spiny neurons in the striatum (Gagnon et al., 2017). However, the most widespread method to quantify dendrites has been Sholl’s analysis (Sholl, 1953), in which concentric rings emanating from the soma are used to calculate the number of branches, branch geometry, and overall branching patterns over distance. For example, Sholl analysis revealed significant reductions in morphological complexity of both the basal and apical arbors of neurons from the dorsal CA1 hippocampus in late-onset Alzheimer’s disease knockout mice compared with wildtype controls (Mehder et al., 2020). In contrast, the morphology of ventral hippocampus and sensory cortex neurons were only minimally reduced in the knockout mice, demonstrating the specificity of Alzheimer’s pathology to particular neuronal types and regions within the central nervous system (Braak and Del Tredici, 2015). Sholl analysis has also been used to show decreased dendritic length and radial dendritic complexity of striatal spiny neurons derived from patients with Huntington’s disease and treated with progerin to induce age-related changes (Machiela et al., 2020).

Although Sholl analysis is an established and widely used method for evaluating neuronal morphologies, it has a number of issues and limitations. Although some 3-dimensional Sholl methods exist (Ruthazer and Cline, 2002; Binley et al., 2014; Gobran et al., 2020), Sholl analysis is typically performed using a 2-dimensional array of concentric rings positioned over a 2-dimensional tracing of a neuron. Therefore, distance in the z-plane is discounted and two branching points within the same Sholl ring can be far apart in the z-direction. Moreover, the output of Sholl analysis is complicated by the fact that it reveals differences by ring distance, which cannot be condensed into a single metric. This makes comparisons between different groups of neurons challenging, although we point out that recent work demonstrates how the Sholl output can be broken down into a branching statistic more amenable for comparing across groups (Bird and Cuntz, 2019). Sholl analysis usually only measures dendritic complexity over a single scale (the ring size, which is normally 10 or 20 μm), although comparisons across several scales are possible by comparing a neuron’s entire arbor with subregions of the arbor (Kutzing et al., 2010).

In contrast to these limitations, fractal geometry may be better suited for characterizing natural phenomena such as neuronal morphology as it allows a 3-dimensional measure of the neuron over multiple scales and is capable of generating a single metric of complexity (Smith et al., 2021). Indeed, fractal methods have already been used in a diverse range of neurobiology investigations. For example, the ability to grow electrodes using nanocluster deposition that resemble the shape of the natural neurons has been developed for potential application in retinal implants (Fairbanks et al., 2011; Watterson et al., 2016). Fractal dimension (*D*) serves as a measure of morphological complexity over multiple scales; a high *D* corresponds to patterns with relatively large amounts of fine structure and therefore high complexity. This metric has been used to successfully discriminate populations of spinal cord, cortical, and retinal ganglion neurons (Elston and Zietsch, 2005; Milosević et al., 2005; Jelinek et al., 2011; Puškaš et al., 2015). Moreover, several studies have shown the value of fractal analysis for identifying neuronal pathology. A significant reduction in dendritic complexity of pathological Purkinje cells in the mouse cerebellum was shown using a 3-dimensional fractal analysis (Kim et al., 2011). Fractal dimension has also been used to identify pathology in retinal vasculature after strokes and in a variety of neurodegenerative disorders (Lemmens et al., 2020).

Here, we aim to assess whether fractal analysis is a useful tool for detecting pathology in CA1 pyramidal neurons in the hippocampus of rats that have lesions in the anterior thalamic nuclei (ATN). ATN dysfunction is a recognized cause of diencephalic amnesia and produces a comparable pattern of memory deficits to that caused by hippocampal system injury (Gold and Squire, 2006; Carlesimo et al., 2011; Aggleton et al., 2016). In rats, ATN lesions result in severe spatial memory deficits which are accompanied by functional changes in the hippocampus such as reduced phosphorylated CREB (pCREB) and immediate early gene (IEG) markers such as c-Fos (Jenkins et al., 2002; Dumont et al., 2012; Dupire et al., 2013; Dalrymple-Alford et al., 2015).

The current investigation will build on previous experiments by some of the authors showing that ATN lesions reduced basal and apical spine density in hippocampal CA1 neurons (Harland et al., 2014). In this same study, groups of ATN-lesion and sham-operated rats (SH) were either housed post-operatively in standard group cages (STD) or exposed to an enriched environment (ENR); the latter used a larger home-cage, additional cage-mates and different daily combinations of objects. A month of environmental enrichment ameliorated spatial memory performance as well as basal and apical CA1 spine density in ATN-lesioned rats to levels comparable to that of the standard-housed controls. Moreover, we found that sham rats exposed to the enriched environment also had increased CA1 spine density compared with standard-housed shams. However, it is unclear whether enrichment will result in increased CA1 dendritic complexity in ATN-lesioned rats.

Other researchers examining the effects of enrichment in intact animals have reported that increases in CA1 spine density are accompanied by increased branching and length of CA1 hippocampal neurons (Faherty et al., 2003; Kozorovitskiy et al., 2005). Both subicular and basal forebrain lesions resulted in a reduction in the complexity of hippocampal CA1 neurons but subsequent enrichment resulted in a reversal of these deficits in only the subicular lesioned rats (Dhanushkodi et al., 2007; Fréchette et al., 2009). Therefore, we would expect ATN lesions to reduce CA1 neuronal complexity because the ATN is connected to the hippocampus *via* the subicular complex and retrosplenial cortex (Shibata, 1993).

Researchers have also shown that spatial memory training (TR) can result in increased dendritic spines in the hippocampal CA1 (González-Ramírez et al., 2014). In the current study, we will include rats that were exposed to spatial memory training as well as pseudo-training (PS), which provided comparable task-related experience but no explicit training on the spatial memory task. In total, dendritic CA1 complexity will be assessed in six groups of rats: ATN-lesion standard-housed trained (ATN-STD-TR); ATN-lesion enriched trained (ATN-ENR-TR); sham-operated standard-housed trained (SH-STD-TR); sham-operated enriched trained (SH-ENR-TR); sham-operated enriched pseudo-trained (SH-ENR-PS); and ATN-lesion enriched pseudo-trained (ATN-ENR-PS).

Fractal analysis will be employed to determine the structural complexity of neurons in rats taken from these six groups. In a recent study, we related the fractal dimension of CA1 hippocampal neurons, *D*_*A*_, to the optimization of competing functional constraints—the ability of dendrites to reach out and connect to other neurons versus the costs associated with building the dendrites (Smith et al., 2021). Within this model, different neuron types were predicted to have different *D*_*A*_ values depending on the relative importance of connectivity and material cost, with higher *D*_*A*_ values indicating a greater emphasis on connectivity. Here, we hypothesize that pathological states of neurons will affect this fractal optimization and consider whether changes in *D*_*A*_ might therefore be used as a diagnostic tool. Specifically, we aim to examine differences in neuron fractal behavior for each of the six rodent groups specified above and further categorize these groups into those with and without lesions to quantify the factors that impact the optimization process. By relating changes in form to changes in function, our approach will improve on simple pattern characterization techniques if successfully implemented.

## 2 Materials and Methods

### 2.1 Rodents

Eighty PVGc male hooded rats were used (8–10 months old, between 366 and 456 g at surgery). The rats were maintained in reversed 12-h light schedule (8 a.m. to 8 p.m.) in their colony room so that all behavioral testing was conducted during the dark phase when activity levels are higher. Body weights were restricted to 85%–90% of free-feeding weight during testing, with free food access for surgery, recovery, and during subsequent 40-day continuous enrichment period. All protocols in this study conformed to the NIH Guide for the Care and Use of Laboratory Animals and were approved by the Animal Ethics Committee, University of Canterbury.

### 2.2 Lesion Surgery

All rats were housed in groups of 3 or 4 per standard plastic cage (50 cm × 30 cm × 23 cm high) before surgery. Based on preoperative spatial working memory performance in a cross-maze, matched pairs of rats were randomly assigned to either the anterior thalamic nuclei (ATN) or sham (SH) lesion group. Rats were anesthetized intraperitoneally with ketamine (70 mg/kg) and domitor (0.5 mg/kg) and placed in a stereotaxic frame with atraumatic ear bars (Kopf, Tujunga, CA, United States) and the incisor bar −7.5 mm below the interaural line. Two infusion sites in each hemisphere were directed at the anteroventral nucleus (AV), followed by a single infusion in each hemisphere directed at the anteromedial nucleus (AM). Each surgery used 1 of 5 anterior-posterior coordinates relative to an individual rat’s bregma to lambda (B-L) distance. For the AV lesions, the AP coordinates from bregma were: −2.4 for B-L ≤ 6.4; −2.45 for B-L = 6.5 to 6.8; −2.5 for B-L = 6.9 to 7.2; −2.55 for B-L ≥ 7.3. The AV infusions were made at ventrality −5.63 mm followed by −5.73 from dura at ±1.52 mm lateral to the midline. The AM infusion was placed 0.1 mm more anterior than the AV sites, with laterality ±1.20 mm and ventrality −5.86 mm. A microinfusion pump (Stoelting, Reno, NV, United States) delivered either 0.16 μl (each of the two AV sites per hemisphere) or 0.20 μl (single AM site) of 0.15 M *N*-methyl-D-aspartic acid (NMDA; Sigma, Castle Hill, NSW, Australia) in phosphate buffer (pH 7.2) at 0.04 μl/min using a 1 μl Hamilton syringe. The needle was left *in situ* at each site for 3 min postinfusion. For SH surgeries, the needle was lowered to 1.50 mm above lesion coordinates without infusion.

### 2.3 Housing

All rats were housed individually for 7–11 days to allow recovery from surgery and then were re-caged in groups of 4 or 5 for post-surgery cross-maze testing. Rats then received 40 days of continuous housing in either an enriched environment (ENR) or standard caging (STD) during which no behavioral testing occurred. Rats that continued with standard caging were housed with new cage-mates. Both types of housing included ATN and SH rats within each cage. ENR rats were housed with 11 or 12 new cage-mates in a large wire-mesh cage (85 cm × 60 cm × 30 cm high) with all the enrichment objects and the position of food and water changed daily using a standardized arrangement of objects that differed every day throughout the enrichment period (Harland and Dalrymple-Alford, 2020). Placement of the enrichment cages within the colony room was changed every fourth day. After 40 days of these different housing conditions, the enriched groups were re-housed in groups of 3 or 4 in standard housing with cage-mates from the same enriched environment cage during the day and enriched housing at night (∼6 p.m. to ∼10 a.m.) for the remainder of the experiment. This procedure facilitated behavioral testing and the provision of the daily food ration after testing (∼5 p.m.) when all rats were in standard cages.

### 2.4 Spatial Memory Training

Spatial working memory was tested for rats in the trained (TR) group using a T-maze configuration embedded in a cross-maze, a task that requires the room’s spatial cues to be used to locate the goal. The same apparatus, room, and distal cues were used for pre-surgery, post-surgery, and post-enrichment cross-maze testing. Rats were familiarized and pre-trained on the maze pre-operatively over a week and then re-trained for 1 day at the start of post-surgery and post-enrichment testing.

Before surgery, all rats were trained to criterion (87.5% accuracy over four consecutive sessions) for a minimum of 15 and maximum of 32 sessions. Six trials were conducted per session throughout testing, with each trial consisting of a sample and test run with a ∼5 s intra-trial delay in the start area. In the sample run, the rat was placed in one of the start areas for 8 s before the door was lifted and an arm block forced it to enter either the left or right goal arm pseudo-randomly (Fellows, 1967) for a 0.1 g chocolate reward. In the test run, the rats were held in 1 of the 2 start areas for 8 s again before being allowed a choice of either goal arm (neither arm was blocked) but was only rewarded (with 0.2 g of chocolate) for entering the goal arm that was not visited in the sample run. For both sample and test runs, once a rat entered a goal arm it was given 10 s to eat the chocolate and/or look around. The rat was returned to the home cage in the testing room between trials until all its cage-mates had received their trial, resulting in an inter-trial interval of about 3–4 min.

All rats were retested in the cross-maze for 10 sessions post-surgery to determine the effects of the surgery (lesion or sham) on spatial working memory. All rats were re-tested for another 10 sessions at 1 week post-enrichment, however, pseudo-trained (PS) rats received a different cross-maze test procedure to the TR rats. Each SH-ENR-PS and ATN-ENR-PS rat was yoked to a SH-ENR-TR and ATN-ENR-TR rat, respectively, receiving an identical sample run and reward. Instead of a regular test run, PS rats received a second forced choice (pseudo-test) run which, for any given day, was pseudo-randomly “always left” or “always right” for that day.

After post-enrichment cross-maze testing, all rats also received 4 days of familiarization and pre-training in an 8-arm radial maze for 1 or 2 days. For each training session, the food-wells at 7 of the arms were baited with 0.2 g of chocolate each and 1 arm was never baited (pseudo-randomly varied across rats) to increase the spatial demands of the task. Arms were pseudo-randomly repositioned at the start of each day’s testing so that the unbaited arm for each rat represented a fixed location in the room. In any session, the rat was removed once all seven chocolate rewards were claimed or allowed a maximum of 20 arm visits or 10 min in the maze. At the start of the session the rat was placed in the central hub for a 5 s delay before all the doors were simultaneously lifted. Once the rat entered an arm, all other doors were closed while the rat ran to the food well and then returned to the central hub, followed by closing that door and another 5 s delay. Testing was conducted on consecutive days for 7 days a week. The task was run for a minimum of 15 days and a maximum of 35 days, or until the rat reached a criterion of 2 out of 3 sessions without visiting the unbaited arm and had made no more than 5 working memory errors in total over the 3 sessions.

The SH-ENR-PS and ATN-ENR-PS rats were pseudo-randomly designated single arm locations (not a physical maze arm). Their procedure matched the other rats, except with 10 visits to their designated arm, which was re-baited each time. For a more detailed description of the spatial working memory tests described above and the apparatuses involved, see the previous study (Harland et al., 2014).

### 2.5 Histology and Model Reconstruction

The rats were euthanized with sodium pentobarbital 24 h after completion of the radial-arm maze task. The brains were removed fresh without perfusion and a 3 mm thick slab encompassing the anterior thalamic region was postfixed in 4% 0.1 M paraformaldehyde and cut into 50 μm coronal sections using a vibratome (Campden Instruments, London, United Kingdom). Cresyl violet staining was used to evaluate the thalamic lesions by an experimenter blinded to group status and behavioral data. The lesion areas were drawn on electronic copies of the Paxinos and Watson rat brain atlas (Paxinos and Watson, 1998) so that automated pixel counts of the damaged regions could be used to estimate lesion volumes by factoring in the distances provided by the atlas. As in previous studies (Mitchell and Dalrymple-Alford, 2006), only rats with lesions that encompassed 50% or more of the ATN and less than 40% damage to the adjacent dorsal medial and lateral thalamic nuclei were included for analysis. Lesion failures occurred in 18 rats, which were not processed further (14 had minor ATN damage; 2 had unilateral lesions; 1 had greater than 40% damage to adjacent lateral thalamic region; 1 had fornix damage). The final number of rats in each group were: 8 in ATN-ENR-PS, 9 in ATN-ENR-TR, 12 in ATN-STD-TR, 9 in SH-ENR-PS, 14 in SH-ENR-TR, and 10 in SH-STD-TR.

Additionally, a 4 mm block containing the hippocampus was cut in the coronal plane and processed with a metallic Golgi-Cox stain, which stains 1%–5% of neurons so that their cell bodies and dendritic trees can be visualized. 200 μm thick coronal brain sections spanning the bilateral dorsal hippocampus were taken from these tissue blocks using a vibratome. A Leica laser scanning confocal microscope (model SP5, Wetzlar, Germany) was used to collect high-resolution image stacks from these coronal brain sections. The image stacks were captured using a 20× glycerol objective lens with a 0.7 numerical aperture, providing an x and y resolution of 0.4 μm. The step size (z distance between image stacks) was 2 μm. Dendritic arbors were manually traced through the image stacks using Neurolucida38 (MBF Bioscience, Williston, VT, United States) and reconstructed into 3-dimensional models comprised of a large set of connected hollow conical cylinders. The models were then exported to the Wavefront obj format. The analysis of these models was done by authors of this study that were blinded to rat ID numbers.

### 2.6 Arbor Radius Calculation

In this study, we define the arbor radius, *R*_*A*_, of a neuron as its radius of gyration. This can be measured as the root mean square distance between any two points on the arbor (Caserta et al., 1995). However, for reconstructions of arbors in which the lengths of the dendritic segments (i.e., the cylinders) comprising the arbor are not uniform, *R*_*A*_ can also be calculated as

$$R_{A}^{2}=\frac{1}{L_{T}^{2}}\overset{K}{\underset{i=1}{\sum }}\overset{K}{\underset{j=1}{\sum }}\delta l_{i}\delta l_{j}{\left(r_{i}-r_{j}\right)}^{2}$$

where $L_{T}$ is the total length of all the dendrites within the arbor, $\delta l_{i}$ is the length of dendritic segment i , $r_{i}$ is the position vector of dendritic segment i , and K is the total number of dendritic segments (Wen et al., 2009).

### 2.7 Modified Sholl and Cumulative Mass Analyses

Traditionally, Sholl analyses of neurons are performed by counting the number of intersections of dendritic branches with concentric rings (in 2 dimensions) or spheres (in 3 dimensions) of increasing radii centered at a neuron’s soma. In this study, we developed MATLAB code that employs a modified version of a traditional 3-dimensional Sholl analysis that calculates the number of intersections, *N*_*I*_, of a neuron’s dendritic branches with concentric spheres of increasing radii, *r*, averaged across spheres centered at 25 randomly selected locations on a neuron’s arbor within a distance of *R*_*A*_/ $\sqrt{2}$ of the center of mass of the arbor (Wen et al., 2009). This sampling of many local origins rather than just one origin centered on the soma accommodates potential variations arising from some parts of the neurons possessing different structural qualities than others. Restricting the selection of local origins to be within *R*_*A*_/ $\sqrt{2}$ of the center of mass of the arbor reduces the number of spheres that have large portions extending beyond the arbor’s periphery. This approach also allows alignment with the cumulative mass fractal analysis which similarly samples many locations (Caserta et al., 1995).

To perform a cumulative mass fractal analysis (also referred to as the mass-radius method) of a neuron’s arbor, we developed MATLAB code that calculates *L*_*in*_, the total length of all dendrites within concentric spheres of increasing radii, *r*, averaged across randomly selected sphere centers using the same process as the modified Sholl analysis. The range of *r* examined is also the same as that used in the modified Sholl analysis. For a neuron with fractal branches, the mass dimension, *D*_*M*_, of its arbor can be measured from this cumulative mass analysis using the following relationship (Caserta et al., 1995; Milosević et al., 2005):

$$L_{in}∼r^{D_{M}}$$

*D*_*M*_ is referred to as the mass fractal dimension because it measures the change in cumulated mass of the object as a function of the size of the region considered (relating length to mass assumes that the branch width does not vary substantially, which is a reasonable approximation for the cylindrical segments of our neurons). Thus, the slope of a double logarithmic plot of *L*_*in*_ versus *r* provides a quantitative value of *D*_*M*_. However, once the radius of a sphere reaches a large enough value that the entire arbor is contained within the sphere, *L*_*in*_ will become equal to *L*_*T*_. As such, this power-law scaling only holds over a finite range of *r*. Our selection of the scaling range for the fit was chosen to be consistent with the scaling range examined in the fractal box-counting method developed in our previous work (Smith et al., 2021), which we briefly describe below.

### 2.8 Box-Counting Analysis

Together with the cumulative mass analysis, our study employed a second traditional fractal analysis technique. Specifically, we performed a 3-dimensional box-counting analysis to determine the covering fractal dimension, *D*_*A*_, of the neuron arbors. By placing an arbor into an array of discretized cubes “boxes” with side-length, *L*_*box*_, and counting the number of cubes occupied by the arbor, *N*_*box*_, we can use the following relationship to determine the arbor’s “covering” fractal dimension:

$$N_{box}∼L_{box}^{-D_{A}}$$

Given that an arbor has a limited physical size and that the reconstructions we examined are created with a limited resolution, this power-law scaling will only hold over a finite range. At the fine size scale, we limited *L*_*box*_ to be greater than 2 μm as the median length and width of the dendritic segments comprising our reconstructed models are 2.6 and 1.4 μm, respectively. This helps avoid resolution effects arising from the segment shapes. At the coarse scale, we limited *L*_*box*_ to be less than one fifth of the largest extent of the arbor in the x, y, or z-directions to ensure sufficient counting statistics. Within these limits, a straight line was fitted for all sets of points that range over at least an order of magnitude on the log-log plot of *N*_*box*_ versus *L*_*box*_ and the fit with maximal *R* ^2^ was chosen to measure *D*_*A*_.

### 2.9 Distorting Arbor Morphology

Using the techniques developed in our previous study (Smith et al., 2021), we created distorted versions of the reconstructed neuron arbors by simultaneously adjusting the forking and weaving behavior of the arbor branches. Each branch is defined by the set of connected segments between either two forking points of a dendrite, a forking point and the terminal point of a dendrite, or a forking point and the initial point on the neuron’s soma that a dendrite grows out from. We define the weave angles as the angles between the branch’s consecutive segments and the fork angles as the first weave angle of a branch. By multiplying the set of all fork and weave angles in an arbor by a constant factor *α*, we explore how changes in neuromorphology track with changes in the fractal dimension of an arbor. In the current study, we examined a range of *α* from 0.5 to 2 in steps of 0.25 (Supplementary Figure S1), yielding a total of 1,404 distorted arbors. The limits of this *α* range are motivated by the need to avoid direct overlap between branches within an arbor (a non-physical scenario). As *α* decreases below 0.5 (especially as it approaches 0), the amount of overlap between adjacent branches near forking points increases, and as *α* increases above 2.0 the arbors branches become so tortuous that intersections between separate branches become unavoidable.

### 2.10 Measurement of Connectivity and Material Cost

The competing functional constraints examined in this study are connectivity, *C*, and material cost, *M*, and these were measured using MATLAB code developed in our previous study (Smith et al., 2021). Briefly, to measure *C* we orthogonally projected a neuron’s dendritic arbor onto a 2-dimensional plane from a given viewing angle, uniformly expanded the branch widths of this projection by 2 μm (this accounts for the potential growth of spines in the space around a branch), calculated the profile area of this expanded projection, and then averaged this expanded profile area over all possible viewing angles of the arbor. Finally, we then normalized this averaged area by the surface area of the convex hull containing the arbor (this accounts for variations in absolute size between arbors). We measured *M* by calculating the volume occupied by a neuron’s dendritic arbor (this is a measure of the mass of the arbor under the assumption that the density of its dendrites is constant throughout) and normalizing this by the volume of the convex hull containing the arbor.

### 2.11 Statistical Analysis

All statistical analyses conducted within this study were done using functions available in MATLAB’s Statistics Toolbox. Specifically, when comparing the mean values of a parameter between the six rodent groups, we performed a one-way ANOVA followed by a post-hoc Tukey-Kramer test at the 5% significance level.

## 3 Results

**FIGURE 1:**
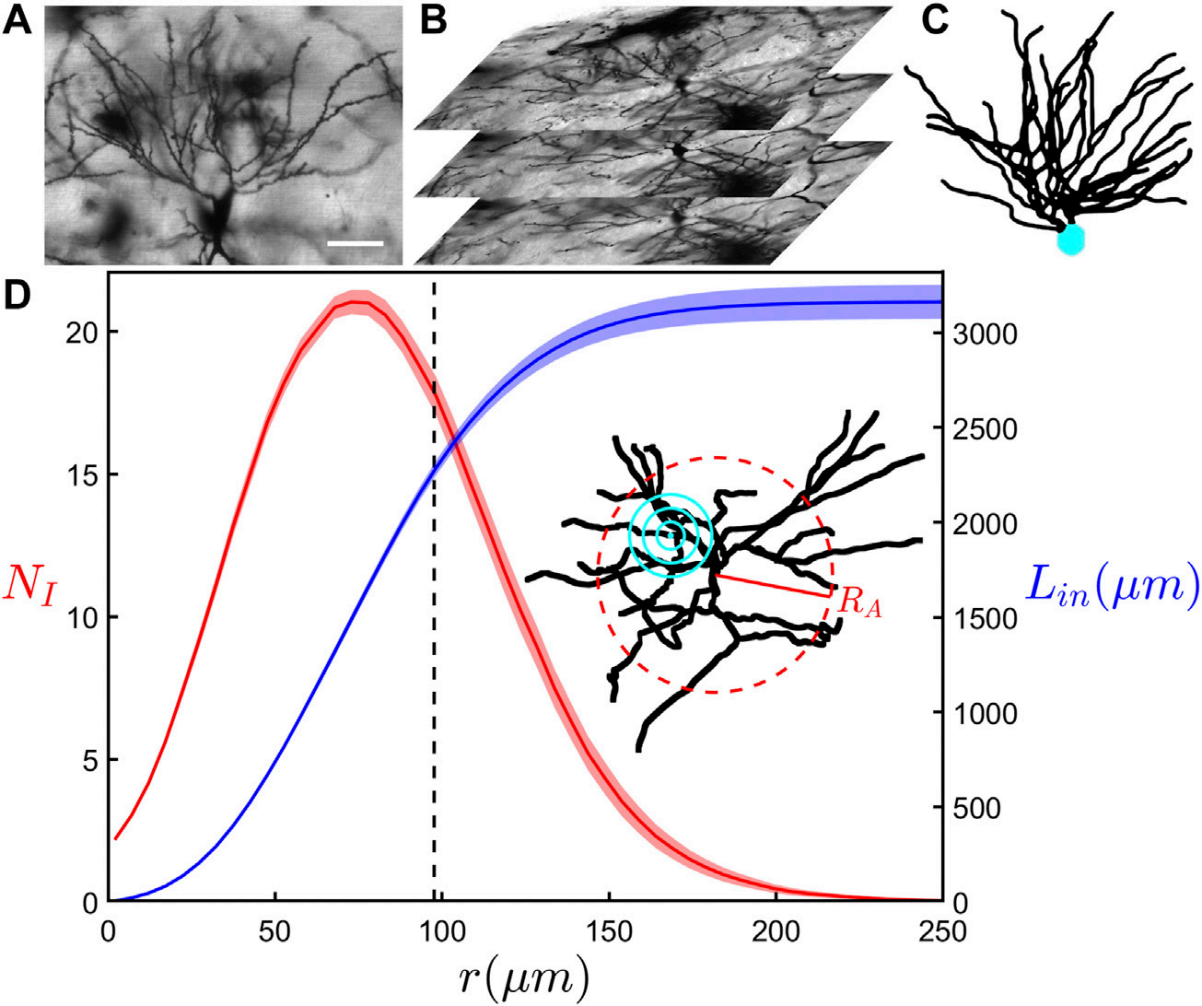
**(A)** A confocal microscopy image in the x-y plane showing the basal portion of a neuron. The scale bar corresponds to 50 μm. **(B)** A z-stack of confocal microscopy images that are used in the reconstruction of the neurons. **(C)** A completed 3-dimensional reconstruction of a neuron with its basal arbor in black and soma in cyan. **(D)** The results of a modified Sholl analysis (red) measuring the average number of intersections of dendrites, *N*_*I*_, with a sphere surface of radius *r*, and a cumulative length analysis (blue) measuring the total length of all dendrites, *L*_*in*_, within a sphere of the same radius. These curves represent the mean behavior across all basal arbors within the SH-ENR-TR group and the shaded region around each curve shows the standard error from the mean. The inset shows an example neuron’s basal arbor with its arbor radius, *R*_*A*_, denoted by the red dashed ring and example sphere radii used in the analyses denoted by the concentric cyan rings. The black dashed line at 98 μm indicates the mean arbor radius within the SH-ENR-TR group. The qualitative behavior of the relationships seen in **(D)** are consistent across all six groups.

Figure 1A shows an example image obtained using confocal microscopy of CA1 pyramidal neurons in the coronal plane of the dorsal rat hippocampus. Figure 1B shows an example z-stack used during reconstruction of a neuron’s arbors. Axonal and dendritic arbors extend from the neuron somas located in the stratum pyramidale of the CA1 region. Although the dendritic arbor features two component arbors (apical and basal), here we focus on the basal arbor (Figures 1A,C) whose complex branching patterns extend into the neighboring stratum oriens where they collect signals from the axons of other neurons. As an indicator of arbor size, the arbor radius, *R*_*A*_, varies between 68 and 134 μm across all examined basal arbors (234 in total), with a mean value of 97 μm. We note that when comparing the mean *R*_*A*_ values across all six rodent groups, no significant difference was found.

**FIGURE 2:**
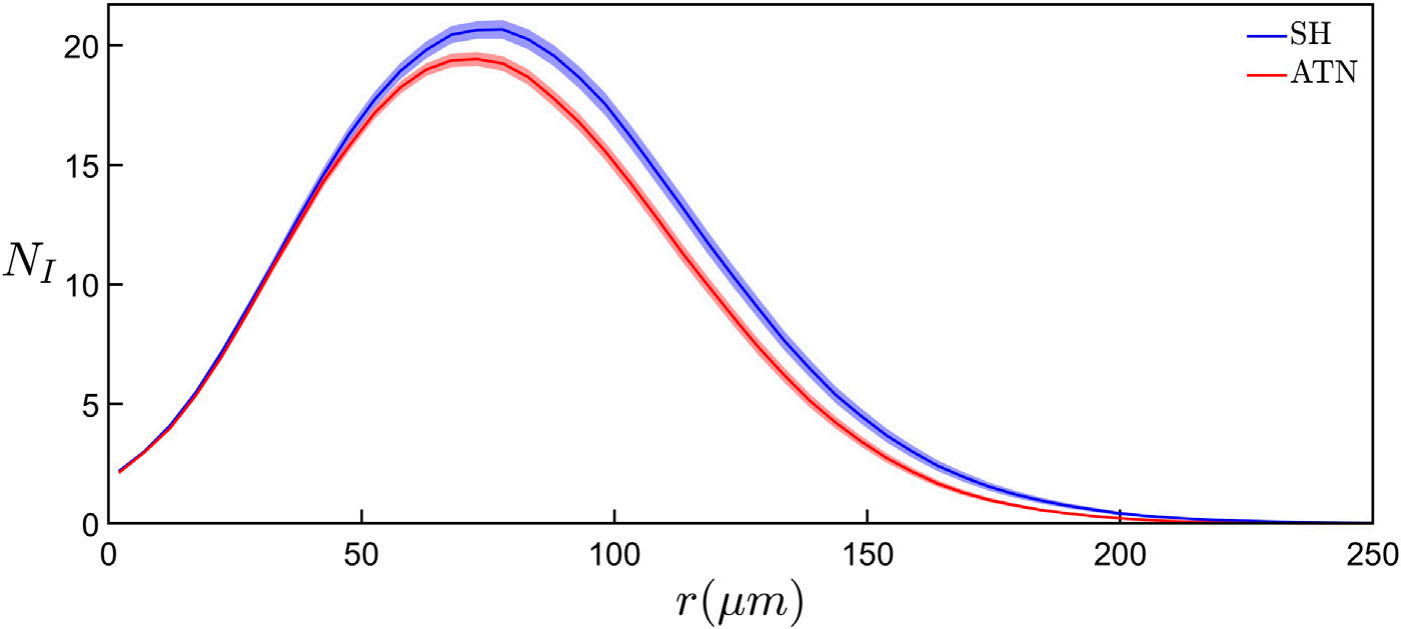
Comparison of the results of modified Sholl analyses showing the average number of intersections of dendrites, *N*_*I*_, with a sphere surface of radius *r* between the basal arbors in the SH (blue curve) and ATN (red curve) categories. Each curve represents the mean behavior across all basal arbors within the corresponding category and the shaded region around each curve shows the standard error from the mean within that category. For *r* ≥ 78 μm, the *N*_*I*_ values of the SH category are significantly higher (*p* ≤ 0.01) than the ATN category.

We begin our investigation by considering the modified version of the traditional Sholl analysis and the cumulative mass analysis. Figure 1D shows the relationship between the average number of dendritic intersections, *N*_*I*_, and the sphere of radius *r* for the SH-ENR-TR group. As *r* increases, we see *N*_*I*_ initially increase to a maximum of 21 at *r* = 73 μm, followed by a decrease as *r* nears the mean arbor radius of 98 μm. While the increase reflects the fractal character of the repeating patterns established by the dendrites (Milosević et al., 2005; Wen et al., 2009), the decrease is a consequence of the measurement technique—it reflects the increased chance of the larger outer sphere surfaces reaching beyond the space occupied by the dendrites. However, its onset can be impacted by any changes in the fractal character towards the neuron periphery. Figure 1D also shows the results of the cumulative mass analysis which charts the total length of dendrites, *L*_*in*_, within a sphere plotted against *r* and reveals a gradual increase that slows in the range of *r* accompanied by the decrease in *N*_*I*_. Equivalent relationships for all six rodent groups can be found in Supplementary Figure S2. We note that the qualitative behavior of these relationships is consistent across all six groups. Interestingly, if we collect the six rodent groups into SH and ATN categories to investigate the impact of lesions, we find that the SH category has significantly higher (*p* ≤ 0.01) *N*_*I*_ values if we consider the Figure 2 data in the range *r* ≥ 78 μm.

**FIGURE 3:**
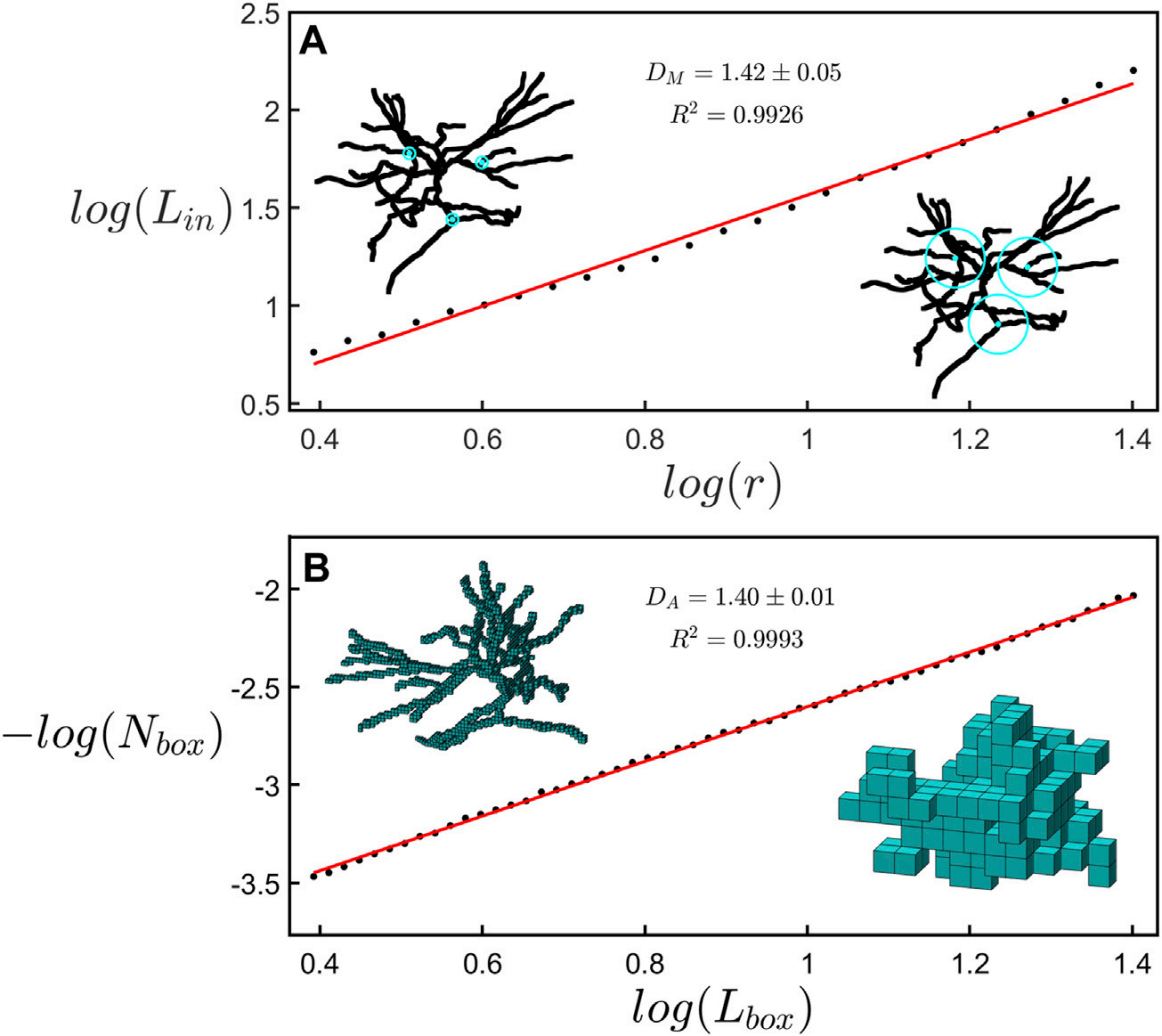
**(A)** A scaling plot of the cumulative length analysis showing the total dendritic length, *L*_*in*_, within a sphere plotted against its radius *r*. The left inset shows rings with a radius of 5 μm at 3 example locations on a neuron while the right inset shows spheres with a radius of 25 μm at the same locations. **(B)** A scaling plot of the number of boxes occupied by a neuron, *N*_*box*_, plotted with respect to the size of the boxes, *L*_*box*_. The left inset shows a representation of the space occupied by a neuron at a box size of 3.1 μm while the right inset shows the same neuron at a box size of 20.3 μm.

In Figure 3, we compare the two methods of fractal analysis employed in this study. Figure 3A shows the measurement of the mass dimension, *D*_*M*_, associated with the cumulative mass analysis, while Figure 3B shows the measurement of covering dimension, *D*_*A*_, associated with the box-counting analysis. The results seen in both panels of Figure 3 correspond to the same neuron. Although the dimension measurements of both methods are in agreement with one another, we note that the results of the linear regression in the box-counting analysis, yielding *D*_*A*_ = 1.40 ± 0.01 (R^2^ = 0.9993), correspond to a better fit than the results of the cumulative mass analysis, yielding *D*_*M*_ = 1.42 ± 0.05 (R^2^ = 0.9926).

**FIGURE 4:**
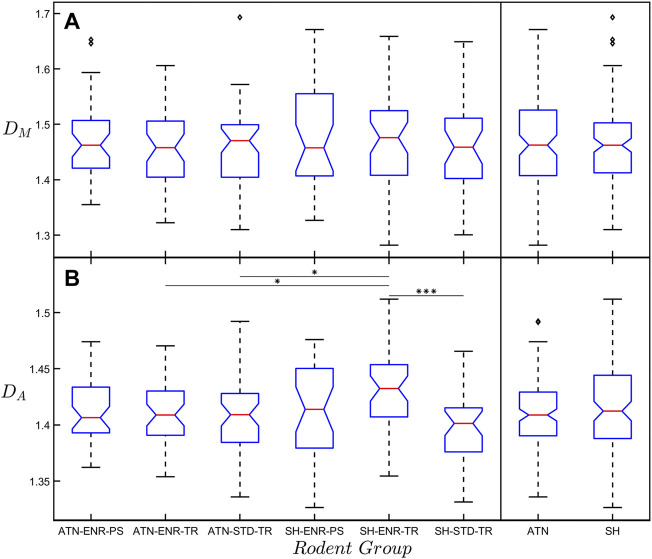
**(A)** Box plot of the mass fractal dimension, *D*_*M*_, with respect to the six rodent groups. The far-right box plots show the results when collecting the six rodent groups into the ATN and SH categories. No significant difference in *D*_*M*_ exists between any of the groups. **(B)** The same as **(A)** but for the arbor fractal dimension, *D*_*A*_. Any significant difference between the SH-ENR-TR group and the other five groups is indicated by the horizontal lines connecting the groups. The diamond-shaped markers seen in some of the groups indicate outliers within that group. The significance markers * and *** correspond to *p* ≤ 0.05 and *p* ≤ 0.001, respectively.

Figure 4 presents box plots of *D*_*M*_ and *D*_*A*_ for the six rodent groups and reports the levels of significance between them. No significant differences are found when comparing mean *D*_*M*_ values across all six groups. However, we find that the mean *D*_*A*_ value of the SH-ENR-TR group is significantly higher when compared to the ATN-ENR-TR (*p* ≤ 0.05), ATN-STD-TR (*p* ≤ 0.05), and SH-STD-TR (*p* ≤ 0.001) groups. Due to our interest in the impact of lesions and also because of the limited amount of data in each group (ATN-ENR-PS: *n* = 41, ATN-ENR-TR: *n* = 41, ATN-STD-TR: *n* = 47, SH-ENR-PS: *n* = 31, SH-ENR-TR: *n* = 42, SH-STD-TR: *n* = 32), we again collect the neurons into the ATN (*n* = 129) and SH (*n* = 105) categories. Interestingly, when comparing the mean *D*_*A*_ value to the mean *D*_*M*_ value for each group or category, the mean *D*_*A*_ value is consistently lower. We note that *D*_*M*_ and *D*_*A*_ belong to a spectrum of dimensions and their magnitudes can be compared using a multi-fractal analysis (Fernández et al., 1999; Jelinek et al., 2013). In our case, the box-counting analysis serves as a more global measure of fractality because it accommodates the whole arbor while the cumulative mass analysis is biased towards the central section (through its restriction of the local sphere centers to be within *R*_*A*_/ $\sqrt{2}$ of the center of mass of the arbor). If the dendrites start to, for example, straighten or fork less towards the arbor periphery, a dimension that measures the whole arbor would be expected to be lower than one that focuses on the central region. Although the differences between *D*_*A*_ and *D*_*M*_ are relatively small for our neurons, based on this potential effect and also on the relative qualities of the associated fits (Figure 3), we will focus on *D*_*A*_ for the following connectivity-cost optimization analysis because its associated parameters similarly quantify the whole arbor.

We stress that the scaling range over which the neuron’s arbor can be described by *D*_*A*_ is limited (typically within 3–30 μm), particularly when compared to mathematical fractals. This is inevitable because the fine and coarse scale limits are influenced by the widths of the branches and the extent of the arbor. However, our previous optimization analysis demonstrated that this scaling behavior is so effective that its limited range is sufficient to optimize the connectivity process (Smith et al., 2021). Nevertheless, because of the narrow scaling range, in Supplementary Figure S3 we confirm the *D*_*A*_ values using an analysis of self-similarity employed in a previous study (Wen et al., 2009) which plots *R*_*A*_ against the total dendritic length, *L*_*T*_, across all the neurons they examined to measure the self-similarity exponent, *μ*. Employing the expressions $R_{A}∼L_{T}^{v}$ and $\mu =\frac{1}{\nu }-1$ , we obtain *μ* = 1.4 ± 0.2 which agrees closely with the mean *D*_*A*_ of 1.41 measured across all arbors we examined.

**FIGURE 5:**
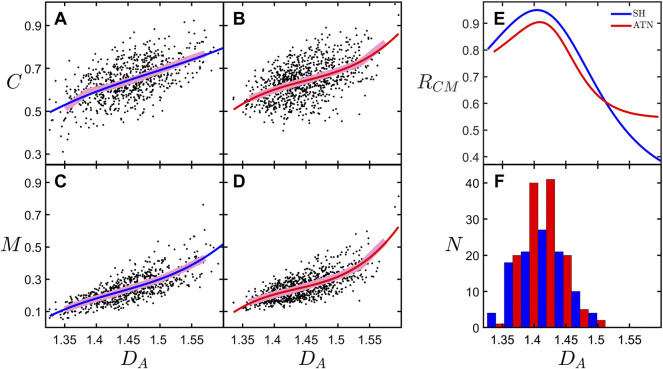
Connectivity, *C*, plotted against arbor fractal dimension, *D*_*A*_, for the **(A)** SH and **(B)** ATN rodent categories. Mass cost, *M*, plotted against arbor fractal dimension, *D*_*A*_, for the **(C)** SH and **(D)** ATN rodent categories. **(E)** The ratio, *R*_*CM*_, of the derivates of the fits of *C* and *M* for each rodent category. **(F)** Histogram showing the number of neurons, *N*, across the range of *D*_*A*_ values for the natural, undistorted basal arbors for the SH and ATN rodent categories. The legend in the upper-right corner of **(E)** applies to all of the figure panels. **(A–D)**, the underlying data corresponds to both natural and distorted basal arbors; the broad, pink lines correspond with binned averages of the data; and the red and blue curves correspond to third degree polynomial fits to the data.

Our previous analysis demonstrated that *D*_*A*_ maps the balance between the neuron’s potential to connect to its neighbors and the associated operational and material costs (Smith et al., 2021). For simplicity, here we will focus on material costs. In Figures 5A,C, we show the dependence of connectivity, *C*, and material cost, *M*, on *D*_*A*_ for the combined SH category. The observed rise in *C* as a function of *D*_*A*_ occurs because the increased amount of fine dendritic structure of high *D*_*A*_ arbors increases their profiles (Materials and Methods Section 2.10). This increase in fine structure also causes a rise in *M*. Figures 5B,D shows the same plots but for the combined ATN category. As was done in our previous analysis, the fits of *C* and *M* versus *D*_*A*_ include both natural and distorted versions of the neurons’ arbors in which we separately adjusted either the forking or the weaving behavior away from the natural state. However, in contrast to the previous study, here we use distorted arbors that have had their forking and weaving behavior simultaneously adjusted away from the natural state. We use this distortion procedure to generate additional “synthetic” arbors for Figures 5A–D. The values of *C* and *M* in Figures 5A–D have been normalized by dividing by the maximum value within the observed range of *D*_*A*_, which is 1.33–1.70 for the SH category and 1.34–1.60 for the ATN category. For clarity, the plotted range of *D*_*A*_ has been limited to 1.32–1.60. This excludes only four outliers within the SH category.

In Figure 5E, we examine the optimization condition by plotting the connectivity-cost measure, *R*_*CM*_, as a function of *D*_*A*_, where we define *R*_*CM*_ by the following equation:

$$R_{CM}=\frac{\left(\frac{dC}{dD_{A}}\right)}{\left(\frac{dM}{dD_{A}}\right)}$$

We note that because *R*_*CM*_ features derivatives, we expect the analysis to be noisy due to sensitivity to scatter in the data. This sensitivity motivates our addition of the distorted arbors in Figures 5A–D as a strategy to increase the number of points in the data set and so improve the fit. This strategy is possible because the distorted neurons follow a similar behavior as the natural arbors (Smith et al., 2021). According to our model, a peak in *R*_*CM*_ is expected to occur at the *D*_*A*_ value at which optimization occurs, *D*_*O*_. The observed peaks in *R*_*CM*_ occur at *D*_*O*_ values of 1.40 and 1.41 for the SH and ATN categories, respectively. Interestingly, in Figure 5F we see that the peaks of the histograms of *D*_*A*_ corresponding to the natural, undistorted basal arbors occur very close to the peaks in *R*_*CM*_, indicating that the majority of neurons in both categories have basal arbors near the optimization condition. However, the differing conditions have not impacted the *D*_*A*_ values at which optimization occurred. Finally, even though we stress the need to categorize because of the low numbers in the individual groups, we do note that the mean *C* value of the SH-ENR-TR group (presumably the group with the three positive conditions) is higher than the other groups, but not significantly so.

## 4 Discussion

Given the central role of neurons as the brain’s “wiring”, our previous research focused on the importance of fractal scaling in establishing connectivity between neurons. *D*_*A*_ was shown to relate to the optimization of competing functional constraints—the ability of dendrites to reach out and connect to other neurons versus the costs associated with doing so. Within this model, different neuron types were predicted to have different *D*_*A*_ values depending on the relative importance of connectivity and material cost with higher *D*_*A*_ values indicating a greater emphasis on connectivity. In the current investigation, we hypothesize that pathological states of neurons might also affect this fractal optimization and consider whether changes in *D*_*A*_ might therefore be used as a diagnostic tool. This analysis represents an appealing development because it relates form to function rather than relying purely on a pattern characterization.

We have tested this hypothesis by examining six rodent groups which varied in brain condition (whether or not they had lesions in the anterior thalamic nuclei) and experience (their housing environment and experience in a spatial task). The optimization process showed that the neurons optimized their connectivity and material cost: when the neurons were collected into SH and ATN categories, the majority of neurons were found to cluster around the optimized fractal dimension, *D*_*O*_. However, based on previous research, we expected that these conditions might influence the *D*_*A*_ values of the associated neurons. In contrast, we found that the optimization analysis does not reveal any significant change in the *D*_*A*_ value between the two categories, suggesting that the differences induced by lesions in the anterior thalamic nuclei are too small to be detected at the small size scales probed by the fractal analysis (approximately 3–30 μm).

Although our modified Sholl analysis of the SH and ATN categories (Figure 2) does not reveal any differences between them at small size scales (consistent with the fractal analysis), it does highlight a drop in morphological complexity at the large scales for the ATN category, suggesting that most changes are occurring in the arbors’ periphery. This would be consistent with a retraction of the CA1 dendritic tree, which may be related to changes in hippocampal molecular markers of plasticity after ATN lesions (Dumont et al., 2012; Dupire et al., 2013). We also note that in Figure 4B the mean *D*_*A*_ value of the SH-ENR-TR group (nominally the three positive conditions) is significantly higher than most of the other groups indicating that this group has the highest morphological complexity. This is in agreement with previous reports that exposure to environmental enrichment increases hippocampal CA1 dendritic complexity (Faherty et al., 2003; Kozorovitskiy et al., 2005). In contrast, the mean *D*_*M*_ value has no significant differences between any groups. This is consistent with our earlier point that, whereas *D*_*A*_ samples the whole arbor, *D*_*M*_ focuses only on its central region and therefore is likely to be insensitive to deterioration (i.e., a drop in complexity) at the periphery. In summary, the combined results of the Sholl and box-counting analyses suggest that ATN lesions induce a drop in complexity at the arbor’s periphery. According to our model, this drop in complexity should translate to a reduction in connectivity. Unfortunately, we cannot determine how the high complexity of the neurons in the SH-ENR-TR group impacts the optimization measure *R*_*CM*_ because the limited number of neurons prevents us from obtaining equivalent Figure 5 plots for each individual group.

**FIGURE 6:**
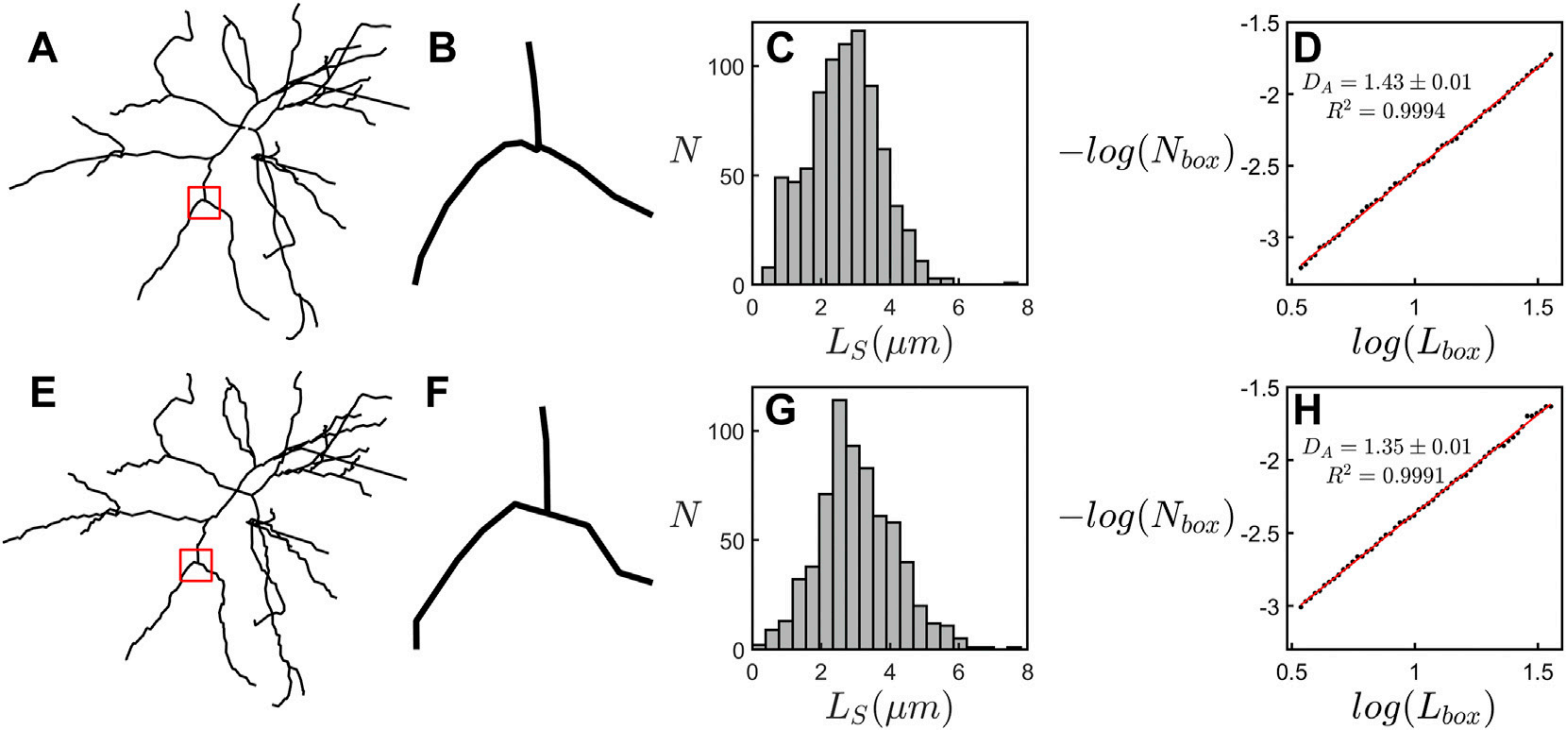
**(A–D)** examine the scaling behavior of an example neuron from ATN-ENR-PS group. **(A)** The example neuron’s basal arbor. **(B)** A magnification of the region specified by the red square in **(A)**. **(C)** A histogram showing the number of dendritic segments, *N*_*S*_, across the range of dendritic segment lengths, *L*_*S*_, for the arbor in **(A)**. **(D)** A scaling plot showing the results of the box-counting fractal analysis of the arbor in **(A)**. **(E–H)** Equivalent panels to **(A–D)** for the arbor after uploading to NeuroMorpho.Org.

It is therefore informative to consider an automated procedure that allows the technique to be applied to publicly accessible repositories, for example online libraries such as NeuroMorpho.Org (Ascoli et al., 2007) with a much larger number of neurons and across a broad range of pathological conditions. We investigated this potential by uploading our models to NeuroMorpho.Org and compared the box-counting fractal analysis presented here to when it is applied to the equivalent uploaded models. Figure 6 provides a visual inspection of an example neuron and its equivalent scaling plots. This inspection reveals a deterioration in resolution, with the NeuroMorpho.Org models appearing more jagged due to a straightening out of the dendritic weave at the finest scales (Figures 6A,B,E,F). This is highlighted in the histograms of dendritic segment lengths, *L*_*S*_, which reveal a drop in the number of segments, *N*_*S*_, at the small scales (Figures 6C,G). This jaggedness impacts the box-count at all scales and results in a drop in *D*_*A*_ (Figures 6D,H) relative to the high-resolution value. As long as we accommodate for this shift in *D*_*A*_ value when using models uploaded to NeuroMorpho.Org, then we can apply the technique presented here to a large range of healthy and pathological neurons in the future.

## Data Availability

The datasets presented in this study can be found in the online repository below: https://github.com/conor-rowland/hippCA1neuronanalysis.
