# Supplementary material for: Investigating Fractal Analysis as a Diagnostic Tool That Probes the Connectivity of Hippocampal Neurons
Source: Front Physiol. 2022 Jun 23;13:932598. doi: 10.3389/fphys.2022.932598 (PMC9260144; doi:10.3389/fphys.2022.932598)
Supplement: Supplementary file 1 [file Table1.DOCX]

**
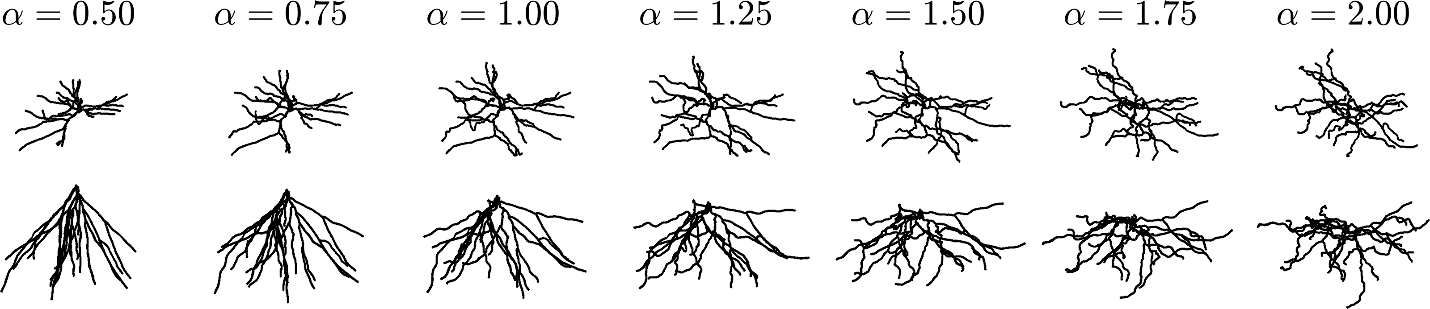
**

**Supplementary Figure 1.** An example of how our distortion technique (that adjusts the forking and weaving behavior of a neuron’s arbor) changes its morphology. The 2 rows of images show the basal arbor of an example neuron from 2 different viewing angles across all of the *α* values used in this study.

**
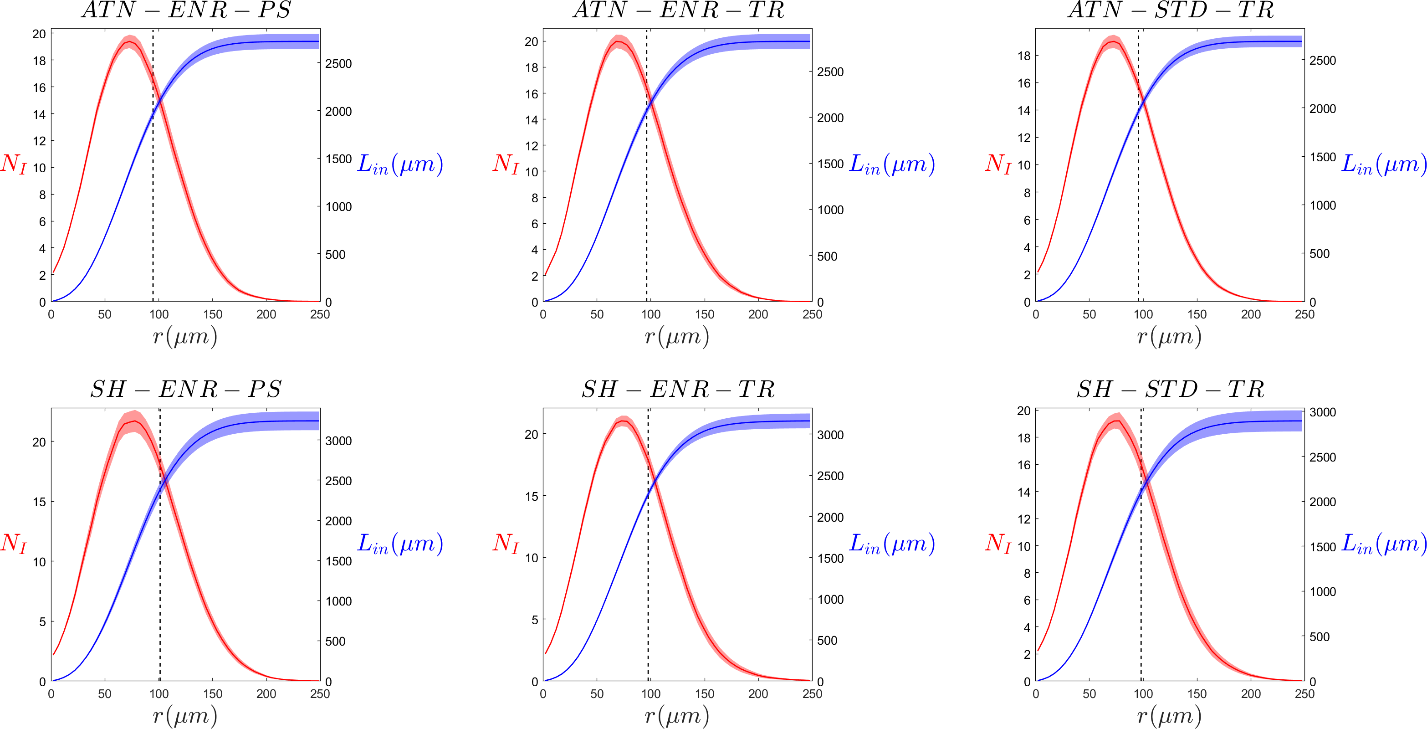
**

**Supplementary Figure 2.** The results across all rodent groups of a modified Sholl analysis (red) measuring the average number of intersections of dendrites, *N_I_*, with a sphere surface of radius *r*, and a cumulative length analysis (blue) measuring the total length of all dendrites, *L_in_*, within a sphere of the same radius. The label above each plot indicates the rodent group examined in that plot. The curves within each plot represent the mean behavior across all basal arbors within the group corresponding to that plot and the shaded region around each curve shows the standard error from the mean within that group. The black dashed lines in each plot indicate the mean arbor radius of the group corresponding to that plot.

**
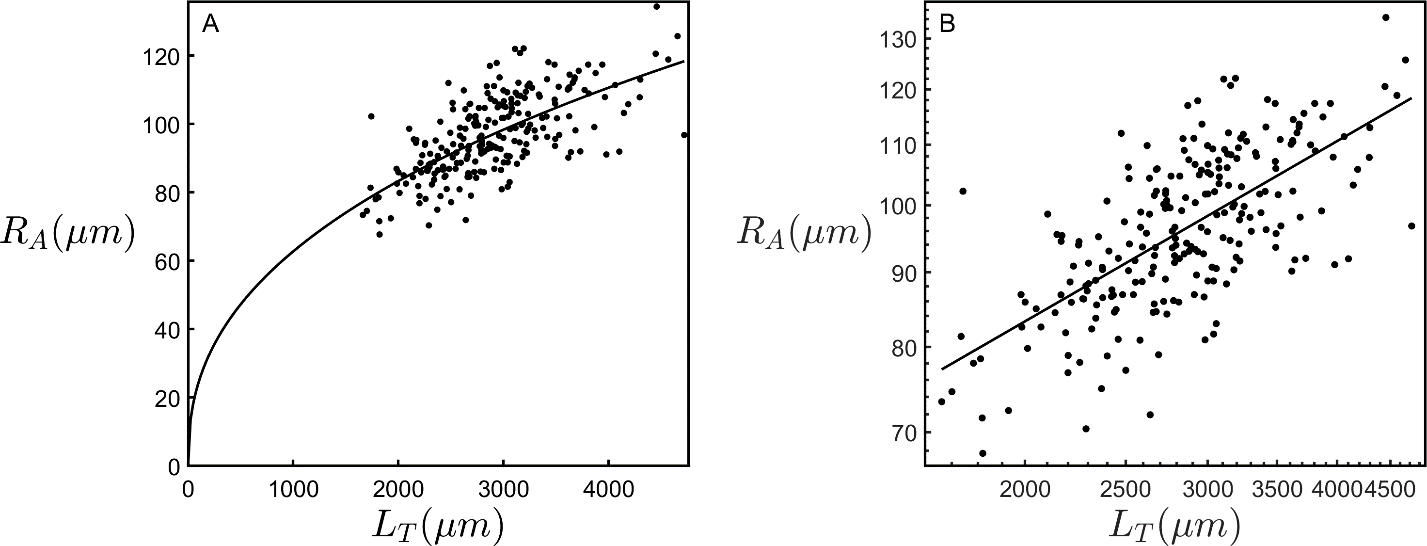
**

**Supplementary Figure 3.** (**A**) A plot of the arbor radius, *R_A_*, against the total dendritic length, *L_T_*, for all examined basal arbors indicating a power-law relationship between *R_A_* and *L_T_*. (**B**) A zoomed in double-logarithmic plot of the same results shown in (**A**). The linear fit in (**B**) is used to determine the scaling exponent, *ν*, in the relationship $R_{A}\sim{L_{T}}^{\nu}$, yielding *ν* = 0.41 ± 0.06.
